# Cortisol and DHEA-S levels in pregnant women with severe anxiety

**DOI:** 10.1186/s12888-020-02788-6

**Published:** 2020-08-05

**Authors:** Philippe Leff-Gelman, Mónica Flores-Ramos, Ariela Edith Ávila Carrasco, Margarita López Martínez, María Fernanda Sarabia Takashima, Fausto Manuel Cruz Coronel, Blanca Farfán Labonne, José Antonio Zorrilla Dosal, Paola Barriguete Chávez-Peón, Saul Garza Morales, Ignacio Camacho-Arroyo

**Affiliations:** 1 Instituto Nacional de Perinatología, 11000 CDMX Mexico City, Mexico; 2 Instituto Nacional de Psiquiatría, 14370 CDMX Mexico City, Mexico; 3 Consejo Nacional de Ciencia y Tecnología/CONACyT, 03940 CDMX Mexico City, Mexico; 4 Hospital General de México, Dr. Eduardo Liceaga, 06720 CDMX Mexico City, Mexico; 5 Unidad de Investigación en Reproducción Humana, Instituto Nacional de Perinatología-Facultad de Química, Universidad Nacional Autónoma de México, 04510 CDMX Mexico City, Mexico

**Keywords:** Steroids, Serum levels, Pregnancy, Anxiety, Cortisol, Cortisol/DHEA-S ratio

## Abstract

**Background:**

A complex interaction between cortisol and dehydroepiandrosterone-sulphate (DHEA-S) is crucial in the stress system balance; several studies have reported increased cortisol levels during chronic stress and a weak counter-regulation by DHEA-S. During pregnancy, scarce information about this system is available, although cortisol and DHEA-S play an important role in the initiation and acceleration of labor. We conducted the present study in order to determine both cortisol and DHEA-S levels during the last trimester of pregnancy in patients exhibiting severe anxiety.

**Methods:**

Pregnant women during the 3rd trimester of pregnancy were evaluated by using the self-reported version of the Hamilton Anxiety Rating Scale (HARS). According to the scores obtained from the psychometric scale, participants were divided into two groups: 1) patients exhibiting a cutoff score > 15 were considered with severe anxiety (ANX) (*n* = 101), and control pregnant subjects (CTRL) (*n* = 44) with a cutoff score < 5. Morning cortisol, DHEA-S and Cortisol/DHEA-S index were measured in all participants. Comparisons between groups were performed; additionally, correlations between clinical variables, biochemical data and HARS were calculated.

**Results:**

Cortisol levels were significantly higher in the ANX group (*p* < 0.001), whereas those of DHEA-S were significantly lower in the same group (*p* < 0.01) when compared to healthy pregnant subjects. An increased cortisol/DHEA-S index was observed in the ANX group (*p* < 0.05). A significant association between cortisol and HARS scores (*p* = 0.03), was observed even after adjusting by gestational weeks (*p* = 0.004).

**Conclusions:**

Our data support that the cortisol/DHEA-S index is higher in pregnant women with high anxiety levels as compared with healthy pregnant women.

## Background

The term ‘anxiety’ may encompass a wide variety of constructs ranging from clinical diagnosis to self-report measures of symptoms to more general measures of stress [1]. Elevated symptoms of anxiety have been reported in 15–16% of pregnant women and in 8–9% of postpartum women [2]. Other reports have shown that the prevalence of prenatal anxiety has been estimated in 25% during the first trimester and 21% during the third trimester of pregnancy [3]. As depression and anxiety are commonly comorbid, anxiety has been considered as a precursor of depressive disorders. It is known that women are more vulnerable than men for developing anxiety disorders [4].

Nonetheless, stress and anxiety are relatively common in pregnant women during the prenatal period, and both disorders affect the mother and the newborn. Effects and consequences of antenatal stress include a diminished capacity for self-care in the mother and therefore, an inadequate nutrition, impact on the gestation and delivery, provoking intrauterine growth restriction^,^ premature births, and low birth weight. The neurodevelopment of children could also be affected by this condition [5, 6]. Interestingly, it has been shown that adverse lifestyle experiences and stressors during pregnancy may trigger high levels of anxiety symptoms [7].

Anxiety during pregnancy is a high-risk factor for developing postpartum depression (PPD). Anxiety itself, may promote several adverse consequences to mother’s mental and physical health [8]. Anxiety and stress during pregnancy are related to fetal heart rate, motor activity [9], preterm delivery [10] and infant behavior [9]. Other risk factors related to prenatal anxiety include increased cortisol levels and pro-inflammatory cytokines that lead to obstetric problems and cesarean section, in addition to the well-known effects produced on the neonate (lower gestational age, prematurity, less insulin-like growth factor in cord blood, less breast-feeding and less self-regulation during the heelstick procedure) [3].

Several studies showed that children from mothers with parental stress and high anxiety are more prone to experience a range of altered physical and physiological outcomes than the children of non-anxious mothers, including a higher risk of developing anxiety and depression, symptoms of attention deficit hyperactivity disorder (ADHD), conduct disorder, and specific regional reductions in brain grey-matter density [3, 11, 12]. Such altered grey matter may be associated with neurodevelopmental and psychiatric disorders, and cognitive and intellectual impairment [12].

Furthermore, several studies have shown that maternal stress and anxiety during pregnancy are associated with neonatal negative outcomes at birthassociated with a reduced score in the Brazelton assessment, in addition to a more difficult temperament [12].

Cortisol represents a key functional biomarker in response to stressors impinging the brain. However, during the perinatal period, the role of cortisol in women displaying affective disorders (anxiety, depression) is yet to be elucidated, due to different methodologies carried out in related studies [13]. The placenta is a neuroendocrine organ that produce and release corticotropin-releasing hormone (CRH). This placental CRH (pCRH) sums to the CRH produced by the maternal hypothalamus [14]. Both pCRH and hypothalamic CRH exhibit a high structural homology, and display a similar-related bioactivities and immunoreactivities. If the role of cortisol is to promote a negative feedback system in the HPA axis; during pregnancy, the cortisol in the maternal compartment enhances the stimulation of CRH in the placenta instead of suppressing it [15].

Women with a well-functioning stress response system may show high levels of plasma cortisol. However, pregnant women may become less responsive to external stressors through a reduced activation of CRH-producing neurons within the parvocellular paraventricular nucleus [15, 16]. This is due to a lower effectivity of brainstem afferents in stimulating CRH neurons (i.e., regarding physical stressors) or from altered processing of limbic structures (i.e., regarding emotional stressors) during pregnancy [16, 17].

In women with a deregulated HPA axis, this attenuation fails to occur and high levels of cortisol secretion that are common in pregnancy could lead to hypercortisolemia [14, 17]. Therefore, hypercortisolemia may increase woman’s risk for developing high levels of anxiety and depressive symptoms [17]. However, withdrawal from an excess level of cortisol during pregnancy may trigger depression in the postpartum period, merely due to an hypocortisolemia response (that is, adrenal secretes less cortisol than the physiologically needed) [18]. The typical pattern of cortisol levels commonly seen in pregnancy involves a gradual increase, peaking at delivery and a sharp decline to baseline level within the first 3 days postpartum [19]. This drop, not only represents the absence of placental cortisol but also reflects a transient suppression of hypothalamic CRH and of pituitary ACTH, respectively [19].

Notwithstanding, under physiological conditions the body normally self-adjusts to the hormone withdrawal in the postpartum period. However, in the presence of PPD, an over-adjustment occurs, leading to hypocortisolemia, precipitating the depressive symptomology [19, 20] through an activation of a cortisol- dependent stimulation of the corticolimbic dopamine neuronal system [21].

Maternal cortisol increases 2 to 4-fold during pregnancy [19, 22]. Cortisol permeates the placenta, which is tightly regulated by the enzyme, 11β-hydroxysteroid dehydrogenase (11 β-HSD2), an enzyme which is highly expressed in the syncytiotrophoblast [23], and which serves as a glucocorticoids barrier, limiting their transfer across the placenta and thereby, preventing the overexposure to these steroids in the fetal compartment [21, 23]. Nonetheless, maternal cortisol accounts for approximately 30–40% of fetal concentrations of cortisol [23].

Glucocorticoids have potent effects on the maturation of several systems and organs [24], most notably, the lung [25] and the brain [17, 24], exerting programming effects on stress-related neural and non-neural systems, producing long-lasting changes in brain structures and enhancing axon and dendrite remodeling in neurons, [17, 26]. However, fetal exposure to excess maternal cortisol results in impaired brain development as a result of neurotoxicity [17, 23, 27].

Prenatal exposure to excess of glucocorticoids impacts on adult pathophysiology [23, 26]. As shown in animal studies, birth weight in rats was shown to be reduced upon prenatal exposure to dexamethasone (a synthetic glucocorticoid agonist) which crosses the placenta [27]. As adults, the offspring exhibit permanent hypertension, hyperglycemic, increased HPA axis activity, increased responses to stressful challenges, and behavior reminiscent of anxiety [23, 26].

Maternal cortisol levels affect both birth and infant outcomes in multiple ways. For instance, cortisol stimulates the synthesis and release of placental CRH [28], which may predict preterm birth [29, 30]. Maternal cortisol also acts directly on the fetus and determines the development of the nervous system [24].

The 3β-hydroxy-5-androsten-17-one, DHEA or dehydroepiandrosterone is secreted by several tissues which include the adrenal cortex, gastrointestinal tract, gonads, and brain. However, the sulfated metabolite, DHEA-S, is the most abundant endogenous circulating steroid hormone [31]. During pregnancy, fetal organs (i.e., adrenal cortex and liver) synthesize corticosteroids and the androgens DHEA, DHEA-S, 16a-hydroxy-DHEA, and the 16a-hydroxy-DHEA-S [32]. Previous studies have shown an interesting association among chronic stress and cortisol, DHEA-S, and the CORTISOL/DHEA-S ratio [33]. DHEA-S has potent anti-inflammatory and anti-glucocorticoid properties [34, 35]. DHEA-S and cortisol exert opposite effects on the response to stress systems and the Cortisol: DHEA-S ratio can be employed to test the impact of both hormones in stress responses [34, 35].

DHEA-S acts as a GABA(A) receptor noncompetitive antagonist and positive allosteric modulator at the NMDA receptor [36]. Moreover, DHEA-S has neuroprotective, antioxidant, antihypertensive, and anti-inflammatory properties [37], among other brain activities (i.e., reduces conditioned fear responding in rodents and humans) [38].

In addition to the steroid-related profile effects, DHEA-S appears to be a potent anxiolytic and antidepressant target [39] and appears to be deregulated in mood and anxiety disorders [40]. Several evidences suggest that increasing concentrations of DHEA-S attenuates anxious and depressive symptomatology, showing that DHEA-S elevations may represent a compensatory response to stress [41]. Anxiety has been highly correlated with cortisol serum levels during the first trimester of pregnancy and the postpartum [42].

Moreover, in animal models, DHEA-S administration showed to reduce depressive- and anxiety-like behaviors. Similarly, in a human three double-blind studies and placebo-controlled studies, DHEA administration in patients exhibiting major depression disorder (MDD) reduced depressive symptoms [43].

Previous studies showed that fetal plasma cortisol levels were increased after 36 gestation weeks (gwk) until active labor [22], whereas DHEA-S levels were increased only at term gestation. However, only the increase in cortisol levels and the higher Cortisol/DHEA-S ratio (stress index) at labor were positively correlated with human parturition [22, 23].

Despite of the studies showing the association between maternal cortisol levels and anxiety levels (i.e., fears, worries about pregnancy) [19, 29]; cortisol and DHEA-S levels as well as the stress index in pregnant women with anxiety are unknown. Thus, in the present paper we describe cortisol, DHEA-S serum levels, and Cortisol/DHEA-S index in pregnant women exhibiting high anxiety levels.

## Methods

### Design of the Study

We conducted a three-year planned-cross-sectional study (2014–2017) similar to the one previously performed to assess the cytokine profile in pregnant women with affective disorders (Leff et al., 2019; 10.1186/s12888-019-2087-6). Thus, pregnant women were invited to participate to study when coming to their gynecology and obstetrics (GO) interview at the Department of (GO) at General Hospital of Mexico (HGM, Dr. Eduardo Liceaga, Mexico City), in addition of pregnant patients who were attending the GO department at the National Institute of Perinatology (INPer). Patients who voluntarily participated in the study, were requested to sign a written informed consent, which was previously reviewed and approved by the Institution Ethical Committee (reference number: HGM, D1/14/112/04/072, 2014–2016).

During the initial interviews at the GO and psychiatry departments, we assessed the eligibility of patients according to the inclusion criteria, in order to collect both clinical, obstetric sociodemographic data and psychiatric characteristics of participants.

### Participants

Pregnant women recruited into the study were attended at the HGM/Prenatal Control Outpatient Unit and at the GO Outpatient Unit at the INPer, respectively. The recruited population comprised pregnant women from 16 to 39 years-old. All patients were coursing a normoevolutive pregnancy between 27 and 39 gwks. A complete clinical evaluation was carried out by the medical staff and an evaluation of sociodemographic variables was done, including marital status, education level and working status. Participants were asked to complete the questionnaires used to measure anxiety and depressive symptoms: Hamilton Depression Rating Scale (HDRS) and the Hamilton Anxiety rating scale (HARS).

Only patients with a cutoff score below 7 points in the HDRS were included. We grouped the participants according to the HARS cutoff scores. Thus, participants who showed a cut-off score < 7 in depression scale (HDRS) and displayed higher scores (> 15) in the anxiety rating scale (HARS) were grouped in the anxious group (ANX). Pregnant women displaying a HDRS score < 7 and a HARS score as < 7 were defined as the control group (CTRL). Elements considered to reject the access to the study (exclusion criteria) were patients exposed or receiving psychotropic medication, subjects with illicit substance use in the last year, patients suffering previous psychiatric diagnosis, obstetric pathologies, acute or chronic infections, and medical diseases such as, neurological, metabolic, cardiovascular, degenerative, immune-related disorders, cancer and/or rheumatic diseases.

Based on our clinical observations, participants displaying high-rating scores of anxious or depressive symptoms were remitted to the psychiatry department for clinical management, all of them were fed back in relation to their affective symptomology.

Blood samples were collected from women who had an overnight fast period and had their psychological status evaluation. A total of 145 participants were included in the study and divided into two groups; *a)* Patients exhibiting anxiety (ANX, *n* = 101) and *b)* healthy pregnant women (CTRL, *n* = 44).

### Anxiety evaluation

Hamilton Anxiety Rating scale [44] assesses the intensity of general anxiety symptoms, such as cognitive, somatic, autonomic symptoms, as well as anxious mood, tension, fear, and insomnia. It is composed by 14 items in a Likert scale. The test has been validated in Spanish, the local language [45, 46]. The standardized scale scores were applied and described as follows: A score from 14 to 17 indicated patients exhibiting moderate to severe anxiety; whereas a score ranging from 17 to 21 signaled patients exhibiting a high intensity anxiety symptoms or severe anxiety (ANX). Scores between 8 and 13 indicated patients with low levels of anxiety and who were excluded from the study. Patients with anxiety scores < 7, were considered as participants without affective symptoms and used as healthy controls in the study.

### Blood sampling

Fast venous blood sampling was carried out under aseptic conditions from 7:00–9:00 am, following standardized procedures for blood extraction and collection. Serum samples were prepared following standard procedures as described (Leff et al., 2019; 10.1186/s12888-019-2087-6).

### Quantification of serum steroids

Cortisol and DHEA-S were measured in duplicates by using a two-step chemiluminescent enzyme immunoassay (IMMULITE 2000 Analyzer System, Siemens USA) following manufacturer instructions. The IMMULITE 2000 analyzer showed a higher throughput (up to 200 tests per hour) when compared to other analyzers [47]. Briefly, 0.5 mL serum samples were used for all assays. Serum samples were incubated with their specific polyclonal anti-steroid antibody followed by colorimetric detection using the enzyme-labeled chemiluminescent substrate, according to manufacturer instructions (IMMULITE 2000). Cortisol and DHEA-S were assayed though the specific LKCO12 (Analytical sensitivity, 5.5 nmol/L) and L2KDS2 (Analytical sensitivity, 0.08 μmol/L) kits, respectively. Calibration ranges used to estimate steroids in serum samples were: Cortisol, 1–50 μg/dL, and DHEAS, 15–1000 μg/dL. Intra-assay covariance was < 7.0%, inter-assay covariance was < 5.0%.

### Statistical analysis

Steroid concentrations are presented as mean ± SEM. Shapiro-Wilk normality test was used to evaluate the distribution of clinical and biological parameters described in the study. The parametric t-test was used to determine the differences between the mean values of serum steroid concentrations between the tested groups. ANOVA with post hoc Tukey test was used to determine the differences in the Cortisol/DHEA-S indexes at the gwk-related time-points during the third trimester of pregnancy. Pearson bilateral correlation analysis was used to detect the correlations between the steroid concentrations and clinical variables used in the study. Similarly, partial correlation analysis was used to estimate the significant correlations between serum steroid levels and clinical variables evaluated in the study, after controlling for gwk. Comparisons between parameters described herein were performed using the non-parametric Mann-Whitney U test. Statistical analyses were performed using GraphPad Prism 7 (GraphPad Softwares Inc. USA) and SPSS software v.24.0 (Armonk, NY: IBM Corp). For all the statistical analysis, the *p* value ≤0.05 was considered significant.

## Results

### Demographic characteristics

*Sociodemographic characteristics of the participants recruited into the study (n = 145) are showed in* Table 1. Average age of participants was 26 years (range 16–39 yrs) with a gestation week average of 34.8 (range 27.5–41.0 gwks). Significant differences were observed in age (t test, *p* = 0.001) and HARS (t-test, *p* = 0.001), between the studied groups.

**Fig. 1:**
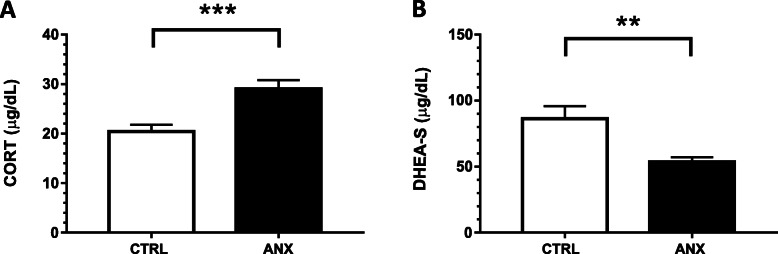
Serum concentration of steroids in pregnant subjects. Figures **a** and **b** depict the serum concentration of adrenal steroids estimated in both CTRL and ANX groups during the 3rd trimester of pregnancy. Steroids were quantified by two step chemiluminescent enzyme immunoassay (see methods). The concentration values of serum steroids are expressed as the mean ± SEM, as described: CTRL; CORT (20.4 ± 1.34 μg/dL), DHEA-S (86.3 ± 9.5 μg/dL). ANX; CORT (29.05 ± 1.8 μg/dL), DHEA-S (53.6 ± 3.6 μg/dL). t-test analysis with Welch’s correction was used to estimate the *p*-values for each steroid assayed in the studied groups. Comparison between serum levels of steroids were performed through the non-parametric Mann-Whitney U test. This test showed significant differences in DHEAS levels (Mann-Whitney U test, *p* < 0.05) and cortisol levels (Mann-Whitney U test, *p* < 0.001), among the studied groups. (*) *p* ≤ 0.05; (**); *p* ≤ 0.001; (***) *p* ≤ 0.0001. Statistical analysis was established at a *p* < 0.05. Abbreviations: ANX, severe anxiety; CTRL; control; CORT, cortisol; DHEA-S, dehydroepiandrosterone-sulphate. Data was calculated using GraphPad v.7

**Table 1:**
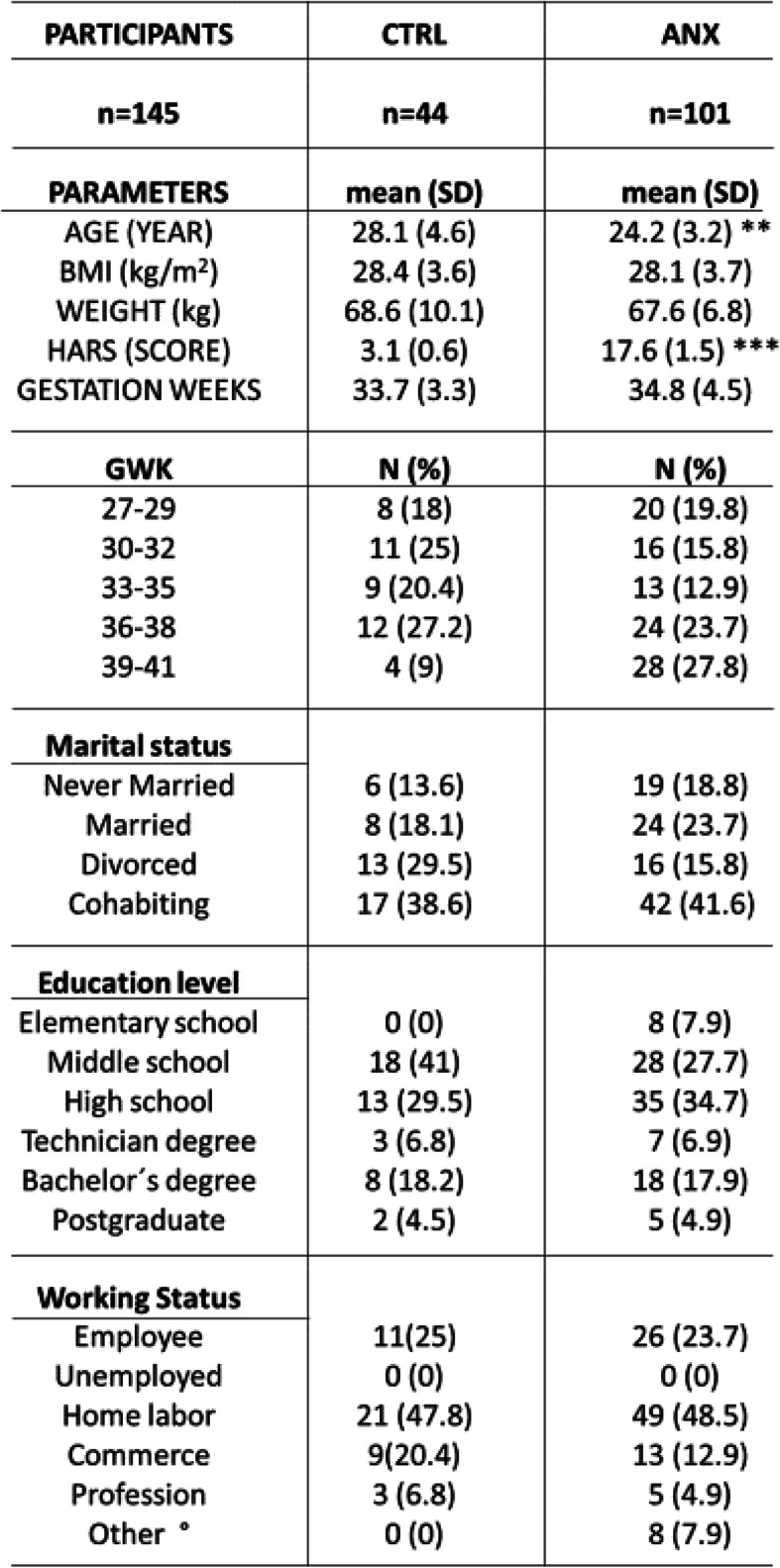
The non-parametric, t-test with Welch’s correction was used to detect statistical differences between demographic measures among the studied groups. Data are expressed as the mean ± SD. (**) *p* < 0.01, indicates the differences in age found among tested groups. (**) *p* < 0.001, indicates the differences found between HARS scores values estimated in participants among the studied groups. (%) Percentages obtained from total subjects in each group. Data were calculated using GraphPad-v.7. Abbreviations: ANX, severe anxiety; CTRL; control; HARS, Hamilton Anxiety Rating Scale; BMI, Body Mass Index; GWK, gestational weeks; CORT, cortisol; DHEA-S, dehydroepiandrosterone-sulphate

### Serum steroids

Figure 1 A-B shows cortisol and DHEA-S concentration in the tested groups. As shown, the ANX group displayed a significant higher serum concentration of cortisol, when compared to that in the CTRL group (t-test, *p* = 0.0004). (Fig. 1a). Conversely, DHEA-S showed a significantly lower serum concentration in the ANX group as compared with that in the CTRL group (t test, *p* = 0.001) (Fig. 1b). Shapiro-Wilk normality test was used to evaluate the distribution of the serum levels of both cortisol and DHEA-S among the studied groups. We observed a non-normality distribution of the data. Therefore, we carried out the non-parametric Mann-Whitney U test used to compare the serum levels of the steroids in the tested groups. Significant differences were observed in DHEA-S (Mann-Whitney U test, *p* = 0.012), cortisol (Mann-Whitney U test, *p* = 0.000), and DHEAS-Cortisol index (Mann-Whitney U test, *p* = 0.000) in the studied groups

### Correlations between serum steroids and clinical variables

Correlations between the steroid profile and clinical parameters among tested groups are presented in Table 2. As shown, the ANX group displayed significant positive and negative correlations between age and HARS (r = 0.25, *p* < 0.05) and between age and DHEA-S levels (r = − 0.46, *p* < 0.01), in addition to the correlations found between cortisol levels and HARS scores (r = 0.26, *p* < 0.05). No other correlations were found between remaining clinical variables described in the study and the high levels of anxiety depicted in this group.

**Fig. 2:**
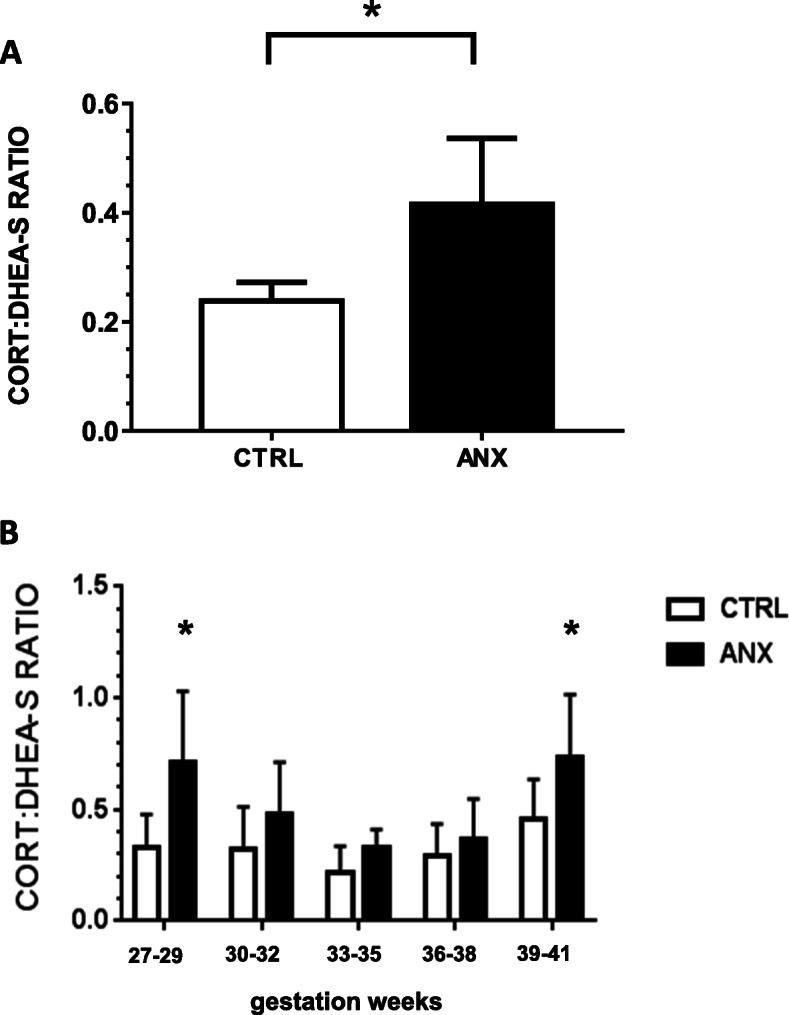
Cortisol: DHEA-S ratios. The figure depicts the ratios estimated between cortisol and DHEA-S serum levels in healthy and pregnant women with severe anxiety. **(a)** shows the estimated cortisol DHEA-S ratio in the tested groups. The ratio values between the Cortisol and DHEA-S serum levels are expressed as the mean ± SD. The ratio values estimated in both CTRL and ANX groups were 0.24 ± 0.03 and 0.41 ± 0.12, respectively. T-test analysis with Welch’s correction was used to estimate the *p*-values between estimated ratios among the studied groups. **(b)** shows the estimated Cortisol: DHEA-S ratio values (described as the mean ± SD) at each of the consecutive gestation week (gwk) periods along the 3rd trimester of pregnancy. The estimated Cortisol: DHEA-S ratios at gwk periods in the CTRL group were: gwk 27–29, 0.71 ± 0.32; gwk 30–32, 0.48 ± 0.23; gwk 33–35, 0.33 ± 0.08; gwk 36–38, 0.37 ± 0.18; gwk 39–41, 0.73 ± 0.28. Similarly, the estimated CORT: DHEA-S ratios at each time-point in the ANX group were: gwk 27–29, 0.33 ± 0.15; gwk 30–32, 0.32 ± 0.19; gwk 33–35, 0.21 ± 0.15; gwk 36–38, 0.29 ± 0.14; gwk 39–41, 0.45 ± 0.18. The mean ± SD values of the gwk time-points (T1-T5) estimated among the tested groups were: T1, 29.0 ± 0.47 gwk; T2, 31.5 ± 0.8 gwk; T3, 34.0. ± 0.8 gwk; T4, 37.4 ± 0.8 gwk; T5, 39.5 ± 0.5 gwk, respectively. Post hoc Tukey test analysis was used to estimate the p-values between CORT: DHEA-S ratios at each time-point among the studied groups. (*) *p* < 0.05. Comparison between serum levels of steroids were performed through the non-parametric Mann-Whitney U test. This test showed significant differences in the DHEAS:Cortisol index (Mann-Whitney U test, *p* < 0.001) among the studied groups. Significant differences were established at a *p* < 0.05. Abbreviations: ANX, severe anxiety; CTRL; control; CORT, cortisol; DHEA-S, dehydroepiandrosterone-sulphate. Data was calculated using GraphPad Prism v.7

**Table 2:**
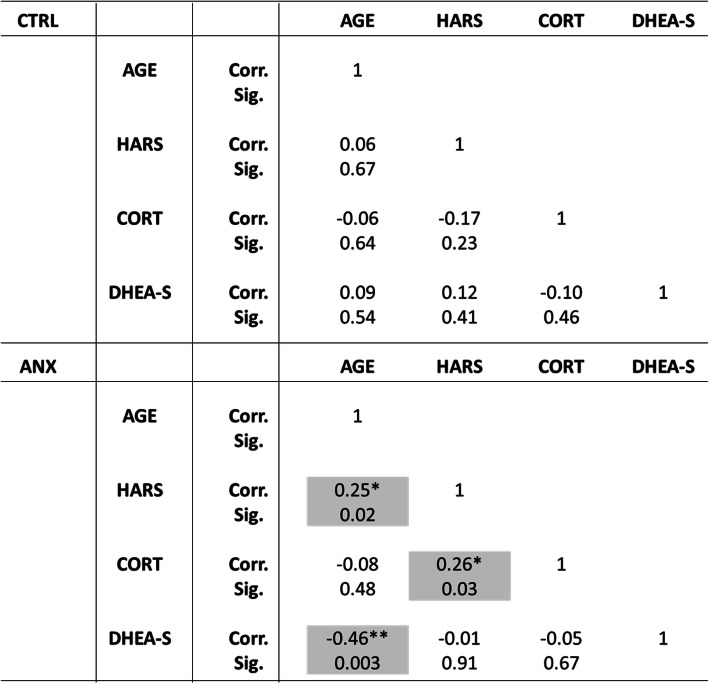
SSPS software v.24.0 was used to determine the Pearson-bilateral correlations among tested groups. Abbreviations: ANX, severe anxiety; CTRL; control; HARS, Hamilton Anxiety Rating Scale; CORT, cortisol; DHEA-S, dehydroepiandrosterone-sulphate; Corr., correlation; Sig., significance. (*) Significant correlation at a *p*-value ≤0.05

Table 3 depicts the partial correlations found between serum steroids, anthropometric and psychometric measures, adjusted by gwks. As shown, a significant positive correlation was found between cortisol levels (CORT) and HARS scores (r = 0.40, *p* < 0.01) in the ANX group.

**Table 3:**
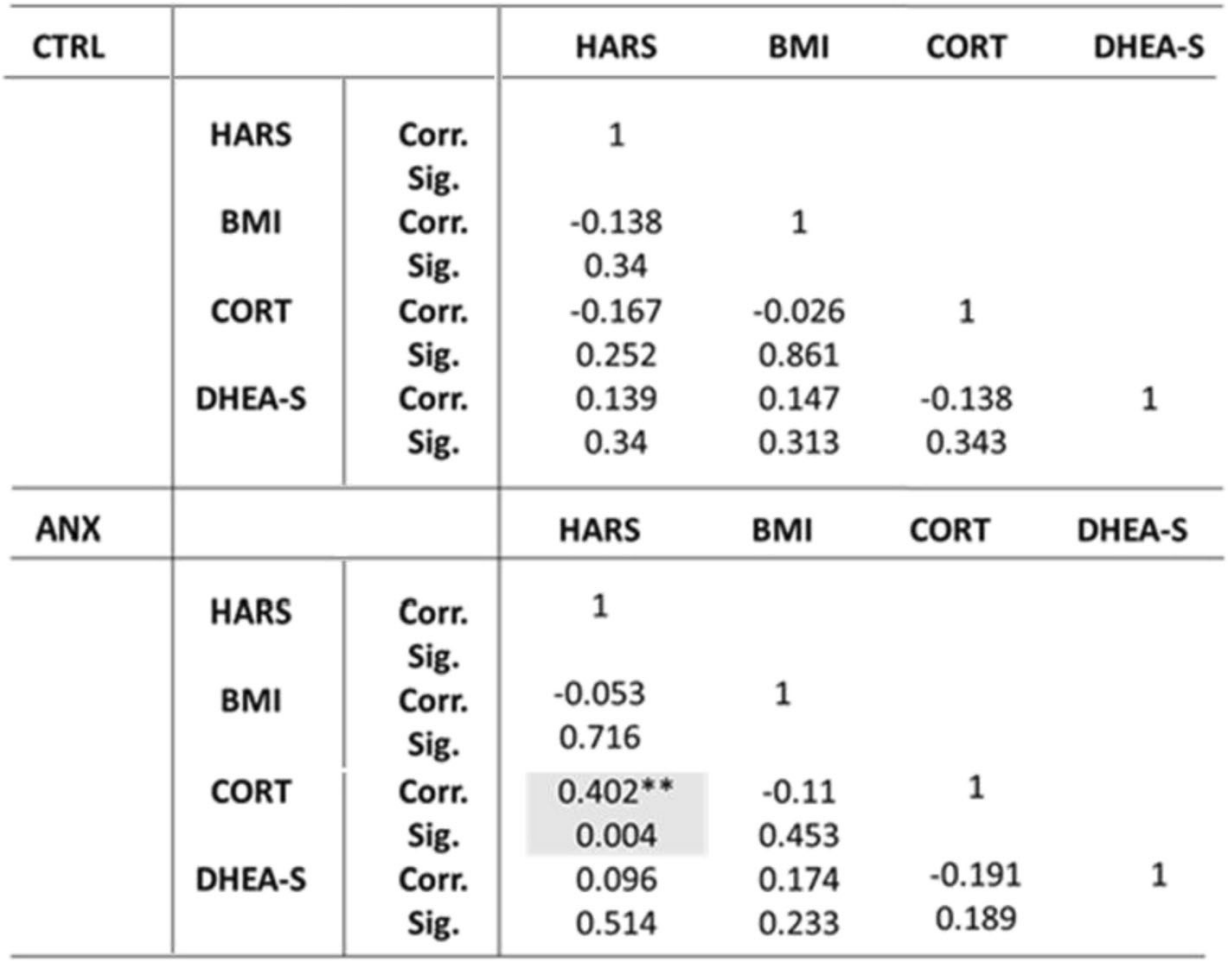
SSPS software v.24.0 was used to determine the Pearson correlations between parameters among tested groups. Correlations and p-values were obtained after controlling variables for gestational weeks, and which was used as the dependent variable in the analysis (see text for details). Abbreviations: ANX, severe anxiety; CTRL; control; HARS, Hamilton Anxiety Rating Scale; BMI, Body Mass Index; CORT, cortisol; DHEA-S, dehydroepiandrosterone-sulphate; Corr., correlation; Sig., significance. (**) Significant correlation at a *p*-value ≤0.01

Table 4 depicts the partial correlations found between serum steroids and clinical parameters adjusted by age. As shown, a significant correlation was observed between cortisol levels (CORT) and HARS scores (r = 0.40, *p* < 0.01) in the ANX group, in addition to a negative correlation observed between gwks and cortisol levels in the CTRL group (r = − 0.32, *p* < 0.05).

**Table 4:**
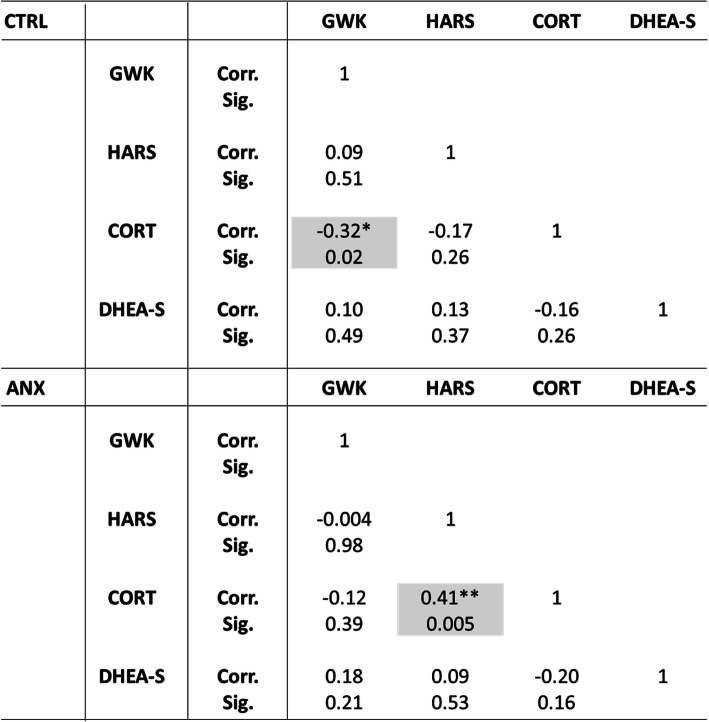
SSPS software v.24.0 was used to determine the Pearson correlations between parameters among tested groups. Correlations and p-values were obtained after controlling variables for age, and which was used as the dependent variable in the analysis (see text for details). Abbreviations: ANX, severe anxiety; CTRL; control; HARS, Hamilton Anxiety Rating Scale; GWK, gestational weeks; CORT, cortisol; DHEA-S, dehydroepiandrosterone-sulphate; Corr., correlation; Sig., significance. (**) Significant correlation at a *p*-value ≤0.01

### Cortisol/DHEA-S index

Figure 2 a-b show the estimated Cortisol/DHEA-S ratios in both the healthy and pregnant women displaying severe anxiety. As shown, the estimated Cortisol/DHEA-S ratio value detected in the ANX group was at least 1.7 times higher than the ratio value estimated in the CTRL group. Significant differences were found between the tested groups (*p* = 0.001) (Fig. 2a). When the steroid ratio values were estimated at different gwks throughout the 3rd trimester of pregnancy, the ANX group displayed significantly higher Cortisol/DHEA-S ratio than the CTRL group, at 27–29 gwks (Tukey, *p* = 0.03) and at 39–41 gwks) (Tukey, *p* = 0.02) (Fig. 2b)

### Trajectory of prenatal anxiety to the postpartum

We explored the postnatal status of two groups of women after childbirth. One group, represented by women with anxiety (*n* = 35; age 23.8 ± 7.6 yrs) and which exhibited high scores for anxiety during late pregnancy (HARS score, 17.6 ± 1.5, Table 1); and a second control group, represented by women (*n* = 26; age 26.2 ± 5.8 yrs) who showed very low scores for anxiety symptoms (HARS score, 3.1 ± 0.6, Table 1) during same gestation period (see methods). Both groups were followed up at 3 days after childbirth (early puerperium) and at 6 wks postpartum (PP) respectively, in order to assess the trajectory of anxiety symptoms from late pregnancy to early postpartum.

Our observations showed that the HARS score values in the group of anxious patients in the early puerperium and at 6 wks PP were, 19.4 ± 1.4 and 20.5 ± 3.0 respectively; whereas in the control group, the anxiety score values obtained were 4.2 ± 1.6 and 4.5 ± 1.4 respectively, at both time-points in the postpartum (Tukey test, *p* < 0.01).

## Discussion

The present study shows that patients exhibiting severe anxiety display an increase in serum cortisol and a decrease in DHEA-S levels, which correlated with the intensity of anxiety symptoms. Previous studies showed a positive correlation between cortisol levels and anxiety symptoms during pregnancy [48, 49], and thus far, our results parallel related studies, showing an increase in cortisol levels during late pregnancy in women with high anxiety.

Previous results from multilevel analysis indicated a robust within-person association between negative mood and cortisol. Such results showed that per each 1.0% increase in negative mood, there was a corresponding 1.9% increase in cortisol concentration. This association was unaffected by advancing gestational age [49].

However, other reports showed that stress and anxiety were significantly associated with subjective feelings of distress, but not with increased cortisol levels in late pregnancy [50]. Such inconsistencies most likely are due to the inclusion in the cited study of depressed patients, while our study included patients with high anxiety levels but not depressed individuals.

Moreover, we cannot exclude some other factors that account for the endocrine results, such as psychometric scale and scores, ethnicity/racial, studied population among others [51–53].

Findings of an abnormal hyperactivity of the stress-response system associated to high baseline cortisol levels and exaggerated response to the dexamethasone/corticotropin releasing hormone test in pregnant women with severe anxiety should be monitored during late pregnancy as indicators of a potential precipitation of either an antenatal and/or a PPD [54].

It has been shown that high levels of anxiety over the course of pregnancy predicted steeper increases in cortisol trajectories compared to lower levels of pregnancy anxiety. This study showed that differences between cortisol trajectories emerged between 30 to 31 weeks of gestation, remaining significant different after adjusting for state anxiety and perceived stress [55].

In addition, DHEA-S has been shown to play a significant role in protection against negative consequences of stress and anxiety-related behaviors, associated to its anti-glucocorticoid effect [37, 39]. Moreover, DHEA-S protects against deleterious effects of cortisol, such as excitatory amino acid– and oxidative stress–induced damage, restores cortisol induced decrements in long-term potentiation, regulates programmed cell death, and promotes neurogenesis in the hippocampus [36, 56]. The anti-glucocorticoid mechanism of DHEA-S has been related to its capacity to interfere with the nuclear uptake of activated glucocorticoid receptors in the neurons of the hippocampus [57].

Cortisol/DHEA-S ratio represents the balance between catabolic and anabolic activity [58]. Impaired DHEA-S secretion together with an increase in cortisol levels results in a higher exposure of the glucocorticoid to CNS and the immune system [59], enhancing an important cytotoxic over the immunomodulatory effects that glucocorticoids exert on target cells [60]. Similar to our findings, a high Cortisol/DHEA-S ratio has been related to chronic stress [61]. This high steroid-related ratio was found significantly associated to depression [62], cognitive disorders [63] and a reduction in the hippocampal volume [63]. Moreover, these effects showed a stronger relationship with high cortisol/DHEA-S ratios than with individual hormone levels [63].

Moreover, interesting studies showed that maternal cortisol, CRH and the State-Trait Anxiety Inventory (STAI) score significantly increased from 2nd to 3rd trimester [8]. At these trimesters, women with high STAI trait scores (≥40) showed an increase in serum cortisol and CRH concentrations and lower insulin sensitivity index (ISI) values than those with low scores (< 40), suggesting that long- and short- term stress and high anxiety are associated with an enhanced maternal hypothalamic-pituitary-adrenal axis response, in addition to an increased secretion of adrenal cortisol and increased placental CRH secretion [64].

Worth to note is that anxiety can be measured with different clinimetric instruments, and the two most widely used are the (State and trait anxiety inventory) STAI and HARS, both of them have a high sensitivity to detect anxiety symptoms; in addition of showing high correlation when measuring anxiety [65]. Thereby, any person suffering from a psychiatric disorder or under stress will display high scores in both instruments, as both assess anxiety-related symptoms.

Therefore, anxiety scores which measure a variety of subjective feelings, autonomic and somatic symptoms, including cognitive functions and behavioral responses, are highly associated with the activation and arousal of the autonomic nervous system that represents one of the main stress regulatory systems, which plays a critical role in modulating the stress responses [66].

Long-term exposure to chronic stressors leads to a progressive dysfunction of the autonomic nervous system, the sympathetic-adrenal-medullary and HPA networks implicated in stress responses, whose overactivity may lead to the release of excessive stress hormones such as catecholamines and cortisol, among several other bioactive mediators that impinge into the brain, and promote altered changes in neural functions as well as in limbic structures-associated to the emotional responses, as occurs in mood-related disorders, such as anxiety [66].

Interestingly, related studies showed that high levels of anxiety may induce an abnormal function of the HPA axis [67]. This study showed that antenatal maternal anxiety measured by STAI displayed a significant positive correlation with high levels of cortisol, supporting the connection between maternal anxiety and HPA axis deregulation. Thereby, the differences in the CORT/DHEA-S indexes found among our studied groups, corroborate the importance of anxiety symptoms during pregnancy and the dysfunction of HPA axis.

Furthermore, the difference found in the cortisol/DHEA indexes between pregnant women with high anxiety versus pregnant women without anxiety, supports and proves the impact of anxiety during pregnancy, as illustrated by Fan F et al. [68], who demonstrated a clear relationship between anxiety and cortisol measurements. Although these authors did not perform DHEA-S measurements. This anti-glucorticoid steroid was shown to decrease the effects induced by hypercortisolism [38, 39] Thus, DHEA-S measurements carried out in our study contribute to the understanding of anxiety during pregnancy.

High anxiety and stress during pregnancy have been linked to adverse maternal health outcomes [69], postpartum depression [2, 4, 70] and long-lasting negative effects in the offspring in the postnatal life [71]. The anxiety effects on the mother’s health during pregnancy include increased cortisol levels, pro-inflammatory cytokines, obstetric problems (i.e., lower birthweight for gestational age, earlier delivery and pregnancy-induced hypertension) and cesarean section [12, 48, 49, 55].

In line with this, recent studies from our group showed that pregnant women exhibiting high levels of anxiety symptoms and severe depression in the third trimester of pregnancy displayed an increase of different subsets of Th1, Th2, Th17 and Treg-related immune biomarkers [72]. However, in the present study we showed that a similar related population displaying only severe anxiety, but not depression, showed a significant increase in CORT/DHEA-S index when compared to non-anxious pregnant women. As anxiety and depression are strongly co-morbid, and it is hard to disentangle the effects of each one, regarding psychosocial and child outcomes [12]; maternal depression showed a wider effect on different types of child maladjustment than maternal anxiety, which appeared more specific to internalizing difficulties in the child [73].

Regarding maternal anxiety and biological responses, previous studies showed that high anxiety and maternal stress conditions during the fetus prenatal life may interfere in immune and endocrine systems in the offspring postnatal life [3, 74]. Moreover, several pieces of evidence have consistently demonstrated that glucocorticoid receptor function is impaired in anxiety disorders, showing an increase in basal cortisol levels and hyper-responsiveness of the adrenal cortex during psychosocial stress [2, 16, 75].

In addition, studies on cortisol levels and antenatal maternal anxiety associated with early life experiences and depressive symptoms in post-pubertal female adolescents [76] showed that female offspring of highly anxious pregnant women display high and flattened diurnal profile of cortisol secretion, indicating a potential expression of a “pre-disease’ pathway” [77]. This data led authors to suggest that a ‘resetting of the HPA-axis’ setting points by antenatal exposure to maternal anxiety during critical periods, may lead to a hyperactive HPA-axis in the female offspring [67].

Furthermore, in children with 8/9 years, 15% of the anxiety symptoms, measured with a child-report standardized anxiety scale, were explained by prenatal maternal state anxiety at 12–22 gestation period [78]. In addition, maternal anxiety was shown to produce changes in cognitive functions in adolescents [79] and depressive mood behavior in 14–15-year-old teenagers [80]. These data suggest that antenatal maternal anxiety already before birth promotes crucial changes in the neurodevelopmental program in the newborn along the postnatal life [12, 17, 67].

Thus, the finding of high cortisol levels in pregnant women with high anxiety, suggest that such population is highly sensitive to environmental stressors which contributes to the altered changes in the HPA axis reactivity and dopamine systems [2] which ultimately could lead to high levels of anxious symptoms in vulnerable subjects, as shown herein, in our recruited ANX population.

In addition, other reports showed that prenatal and postpartum low levels of DHEA-S in plasma were found associated with higher postpartum ratings of depression [48]. Overall, our findings are of clinical relevance, since high levels of cortisol during pregnancy represent a crucial risk factor that contributes to non-optimal pregnancy outcomes [17, 24] such as preterm birth, neonates with lower gestational age and birthweight, high neonatal mortality, and pediatric health problems [8, 66, 68].

Although recent reports showed that massage therapy combined with group interpersonal psychotherapy showed to be effective in reducing both prenatal and postpartum depression, cortisol levels, prematurity and low birthweight in highly risk pregnant population [79]; several limitations of these studies were outlined in clinical research which requires several predictor variables, such as progesterone/estradiol ratios, immune factors and genetic determinants [79], including measuring cortisol/DHEA-S ratios (proposed herein) in highly risk populations displaying chronic stress and high levels of anxiety, as previously reported for related risk populations [33].

Our data suggest that biomarkers associated with an abnormal dysfunction of the HPA axis are crucially important in vulnerable pregnant women exhibiting chronic stress and high levels of anxiety symptoms [50, 54]. Thus, measuring HPA axis-related biomarkers and CORT/DHEA-S index in pregnant women with high anxiety might be greatly useful in assessing HPA axis dysfunction and if psychological and/or medical interventions are effective in ameliorating affective symptoms in highly risk pregnant population.

## Conclusions and perspectives

Our data show that patients exhibiting severe anxiety display a significant increase in serum cortisol and a significant decrease in DHEA-S compared with healthy pregnant women. In addition, Cortisol/DHEAS ratio correlated with the intensity of anxiety symptoms.

As anxiety and depression are usually comorbid, it is imperative to perform studies to explore further correlations between biological mediators and anxiety symptoms in women at high risk of developing severe depression during mid to late pregnancy.

### Limitations

Several limitations in the present study should be noted. First, we applied the self-reported HDRS and HARS instruments used to evaluate depressive and anxiety symptoms, which have not been extensively used in pregnant women, as compared to other psychological instruments (e.g., the CES-D, EPDS, STAI, and GAD-7). Second, blood sampling was performed at a single time at the entry of participants into the study. Thus, to detect changes in steroid serum concentrations during late pregnancy two specific blood-sampling time-points must have been elected as the optimum in our studied population. Third, we did not evaluate the prevalence of cigarette smoking in recruited participants, an important variable that might have influenced our results. Also, pre-gestational weight could contribute to further information about the relevance of weight gain and BMI associated with the determination of the steroid profile.

## Data Availability

The datasets used and/or analyzed during the current study are available from the corresponding author on reasonable request.

## Abbreviations

gwks: Gestation weeks
HARS: Hamilton Anxiety Rating Scale
HDRS: Hamilton Depression Rating Scale
DSM-5: Diagnostic and Statistical Manual of Mental Disorders
DHEA-S: Dehydroepiandrosterone sulfate
CTRL: Control
